# Informing the Development of a Standardized Clinical Definition of Neonatal Abstinence Syndrome: Protocol for a Modified-Delphi Expert Panel

**DOI:** 10.2196/25387

**Published:** 2021-09-07

**Authors:** Dmitry Khodyakov, Shahla M Jilani, Stephanie Dellva, Laura J Faherty

**Affiliations:** 1 RAND Corporation Santa Monica, CA United States; 2 Pardee RAND Graduate School Santa Monica, CA United States; 3 Office of the Assistant Secretary for Health US Department of Health & Human Services Washington, DC United States; 4 RAND Corporation Boston, MA United States; 5 Boston University School of Medicine Boston, MA United States

**Keywords:** Delphi, ExpertLens, expert panel, neonatal abstinence syndrome, neonatal opioid withdrawal syndrome, neonatal withdrawal, neonatal, neonates, opioid, opioids, withdrawal, infants, clinical, newborn, newborns, perinatal, postnatal

## Abstract

**Background:**

Neonatal abstinence syndrome (NAS) is a postnatal withdrawal syndrome that most commonly results from prenatal opioid exposure. Every 15 minutes, an infant is born in the United States with signs of NAS. The field lacks a standardized clinical definition of NAS, complicating discussions on programmatic and policy development to support opioid-exposed mothers and infants.

**Objective:**

The goal of this paper is to describe a protocol for a systematic expert panel process to inform the development of a clinical definition of NAS.

**Methods:**

We will conduct two three-round online modified-Delphi panels using the ExpertLens system and will follow the recommendations for Conducting and REporting of DElphi Studies (CREDES). One panel will focus on developing key components of a clinical definition of NAS, and the second panel will focus on neonatal opioid withdrawal syndrome (NOWS), which is a term that has come into use to differentiate opioid-exposed infants from infants exposed to other substances in utero. However, there is lack of agreement on the precise clinical definition of NOWS and how it is distinct from or overlaps with NAS. Each panel will complete two rating rounds and a discussion round using a similar protocol. We will analyze all rating data descriptively and determine the presence of agreement within and between the two panels. We will also perform thematic analysis of the qualitative comments to contextualize the panel findings.

**Results:**

The panels were convened between October 29 and December 17, 2020. Their results were disseminated and discussed at a national conference on NAS that took place on March 17-18, 2021.

**Conclusions:**

A standardized clinical definition of NAS will help to better characterize NAS incidence and to design effective clinical, public health, and policy interventions to support opioid-exposed mother-infant dyads.

**International Registered Report Identifier (IRRID):**

DERR1-10.2196/25387

## Introduction

From 2000 to 2016, the United States experienced a seven-fold increase in neonatal abstinence syndrome (NAS) [1,2], also known as neonatal opioid withdrawal syndrome (NOWS) [3,4]. NAS is a postnatal withdrawal syndrome most commonly caused by prenatal opioid exposure [5]. An infant is born every 15 minutes with signs of NAS, and total hospital costs for NAS-related births exceeded US $500 million in 2014 in the United States [2]. The incidence of NAS varies substantially across the states, ranging from 0.7 cases per 1000 live births (Hawaii) to 33.4 per 1000 births (West Virginia) [6,7]. However, these statistics have an important limitation: the field lacks a standardized clinical definition of NAS.

NAS is a heterogenous condition that may result from both maternal nonprescribed opioid use *and* prescribed opioids such as the use of medication for opioid use disorder (eg, methadone and buprenorphine) [5]. NAS may also be associated with other prenatal exposures such as benzodiazepines and nicotine [8]. Moreover, the clinical presentation of NAS is highly variable [9], leading to a lack of consensus around the definition, which has downstream consequences for surveillance [10] and policy efforts. Some infants have mild signs of withdrawal that can be managed with targeted nonpharmacologic interventions, whereas others require multiple medications to control their symptoms during weeks-long hospitalizations [11]. The relationship between maternal opioid dose and NAS severity is also unclear [12-16]. Given its unpredictability and variable presentation, there is a need to develop a standardized definition of NAS to accurately characterize the burden of this public health challenge, and consequently design effective clinical, public health, and policy interventions to support opioid-exposed mother-infant dyads.

To address this need, the U.S. Department of Health and Human Services (HHS) contracted with the RAND Corporation, a nonprofit research institution, to engage national experts in a rigorous process to develop a standardized clinical definition of NAS. This work contributes to a multipronged HHS initiative on NAS [17]. Engaging experts with a range of expertise is both critical and appropriate to defining this complex condition because there is no consensus around its clinical definition. Here, we describe our protocol for the systematic online engagement of national experts to provide input to inform the development of a standardized clinical definition of NAS.

## Methods

### Design

Our approach to expert engagement will consist of two modified-Delphi expert panels to explore the presence of agreement around key components of a clinical definition of NAS. The study design was developed in consultation with a six-person advisory board of leading national experts on this topic who have been engaged with the HHS’s initiative on NAS (see Multimedia Appendix 1). We will follow the guidance for Conducting and REporting of DElphi Studies (CREDES) [18].

To facilitate the process of expert engagement, especially during the COVID-19 pandemic, the modified-Delphi panel will be conducted completely online using ExpertLens, a previously evaluated platform for conducting iterative expert elicitation and stakeholder engagement panels [19-21]. Instead of traveling to a centralized location for an in-person meeting, ExpertLens participants provide answers to close-ended and open-ended questions, review automatically generated reports comparing their responses to close-ended questions with those of other participants, discuss group responses using a moderated discussion board, and revise their answers all from the comfort of their own homes or offices.

### Participant Recruitment

On October 6, 2020, we reached out to 22 national experts on NAS, including neonatologists and general pediatricians, as well as those with expertise in clinical pharmacology and psychiatry, with an invitation to participate in these panels. In our recruitment efforts, we prioritized experts with significant clinical expertise in the care of infants with NAS. Identified experts were contacted via their publicly available email addresses and invited to express their interest in participating in this panel. The invitation email explained the purpose of the study, its funder, and the expected time commitment.

### Panels and Panel Composition

After reviewing the list of all invitees who express interest in participating in this study, the research team will select experts to assemble two panels. One panel will focus on identifying key components of a clinical definition of NAS, and the other panel will define NOWS using a similar protocol.

The panels will be limited to approximately 9 experts as recommended by the RAND/UCLA Appropriateness Method (RAM) manual for conducting clinical expert panels [22]. We will aim for balance between the panels in terms of participants’ professional backgrounds and geographic regions because there may be variation in how NAS is defined across disciplines and around the country. We will also aim to balance panels based on participants’ stated preference for using NAS or NOWS (if known).

### Data Collection

The data collection protocols will be developed based on a literature review performed by HHS and national experts, input from RAND subject matter experts, the HHS NAS initiative’s advisory board, and a pilot test. 

**Figure 1:**
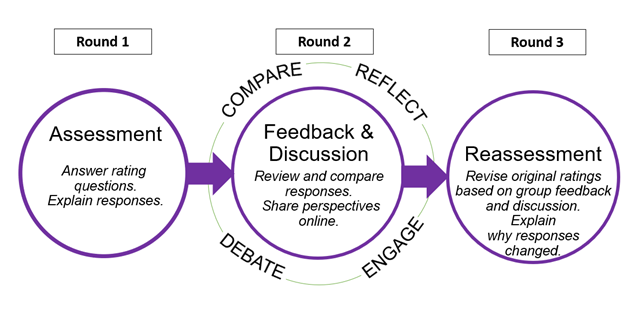
Three-round ExpertLens design.

The data collection began in October-November 2020 and followed a typical modified-Delphi protocol, which includes two rounds of rating with a round of discussion between the two rating rounds (Figure 1) [22-24]. No additional rating rounds will be conducted if agreement is not reached after the final round of ratings.

In Round 1, an assessment round, experts will be instructed to think about a full-term infant in the first week of life with no known medical conditions, and to rate and comment on different pieces of information about the infant and the mother: (1) prenatal exposure to opioids and/or other substances, (2) infant signs of withdrawal from opioids and/or other substances, and (3) toxicology test results (see Textbox 1 for additional details).

**Types of information about mother-infant dyads for panelists to consider in the ExpertLens process.**

**Information about whether or not the infant had prenatal exposure to…**

- opioids alone
- opioids plus other substances (eg, benzodiazepines, selective serotonin reuptake inhibitors [SSRIs], tobacco)
- substances (eg, benzodiazepines, SSRIs, tobacco) but did not have prenatal exposure to opioids

**Information about whether or not the infant…**

- shows signs of opioid withdrawal
- signs of withdrawal from substances other than opioids
- shows dysregulation in at least one domain of infant development such as motor control (eg, hypertonia, tremors) or responses to stimuli (eg, exaggerated Moro reflex)
- requires nonpharmacologic measures to manage withdrawal
- requires medication to treat signs of withdrawal

**Information about whether or not…**

- the infant’s toxicology test is positive for opioids alone
- the infant’s toxicology test is positive for opioids plus other substances
- the infant’s toxicology test is positive for substances other than opioids and is negative for opioids
- the mother’s toxicology test is positive for opioids alone
- the mother’s toxicology test is positive for opioids plus other substances
- the mother’s toxicology test is positive for substances other than opioids and is negative for opioids

To provide their input on each piece of information, participants will use 9-point Likert-type scales to answer the following two questions and explain their ratings: (1) How necessary is this information for distinguishing between infants *with and without NAS [NOWS]*? (2) How helpful is this information for distinguishing between infants *with and without NAS [NOWS]?*

Because of the wide variation in the clinical manifestations of withdrawal in infants, experts will also be asked to use a 9-point Likert-type scale to respond to the following question that will provide input on 10 common clinical signs of withdrawal as described by Gomez-Pomar et al [25]: How characteristic is this sign of NAS (NOWS)?

Moreover, participants will provide feedback on an alternative approach to assessing infant withdrawal that looks for dysregulation in four domains of infant functioning, rather than assessing signs and symptoms individually or in combination [26-28]. This approach is intended to give clinicians a holistic understanding of infants with opioid exposure to distinguish between infants with and without NAS or NOWS. Participants will review a brief description of this approach and then use 9-point Likert-type scales to answer the following questions: (1) How different is this approach from the way withdrawal signs are currently assessed in clinical practice? (2) How useful is this approach for assessing opioid withdrawal in an infant? (3) How feasible would it be to use this approach to distinguish between infants with and without NAS (NOWS)?

**Figure 2:**
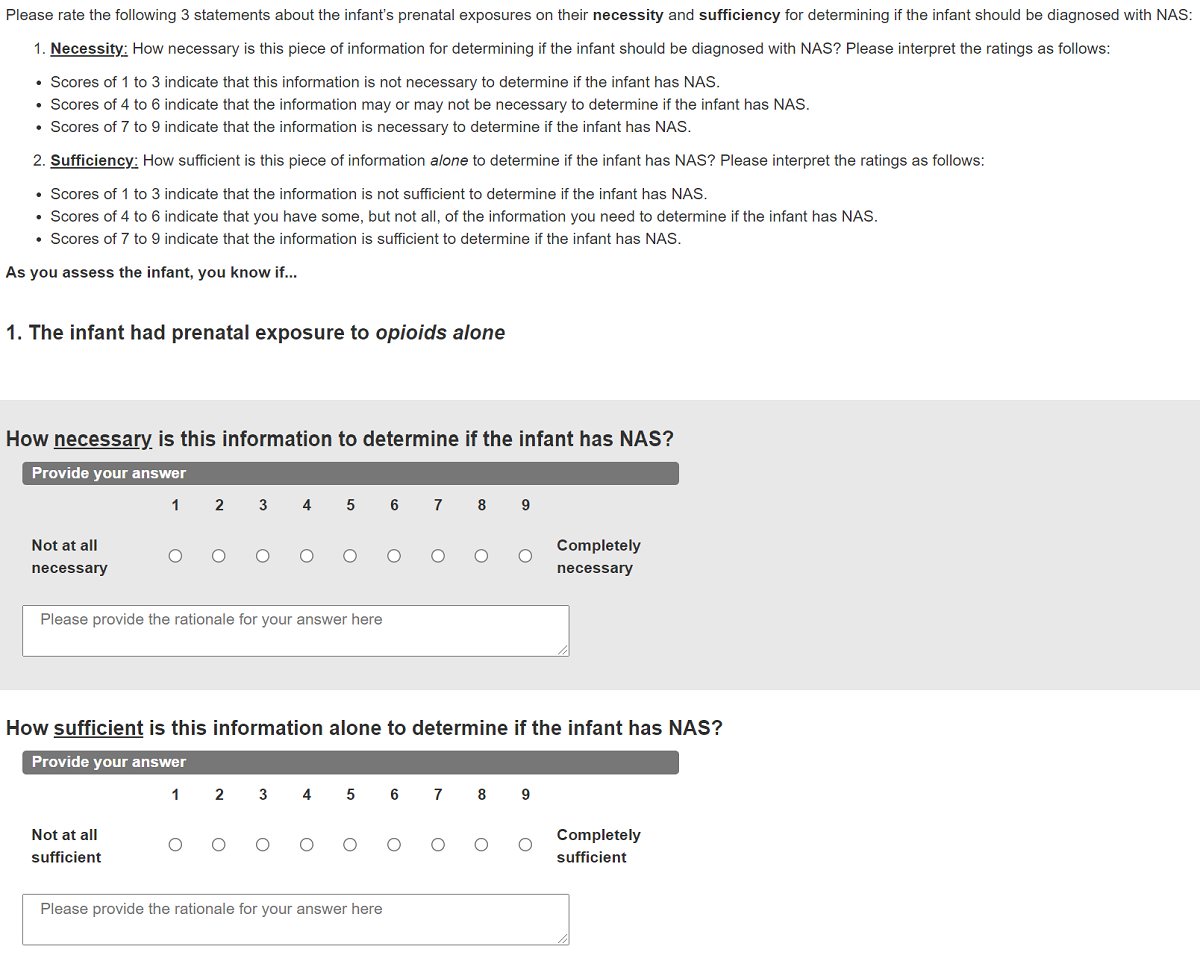
Round 1 mock-up screenshot.

Finally, at the end of Round 1, we will ask participants to provide their suggested clinical definitions of both NAS and NOWS. These final open-ended questions will help to validate the results of our rating process and assess how experts’ definitions may evolve over the course of the study. Figure 2 shows a screenshot for the questionnaire in Round 1.

**Figure 3:**
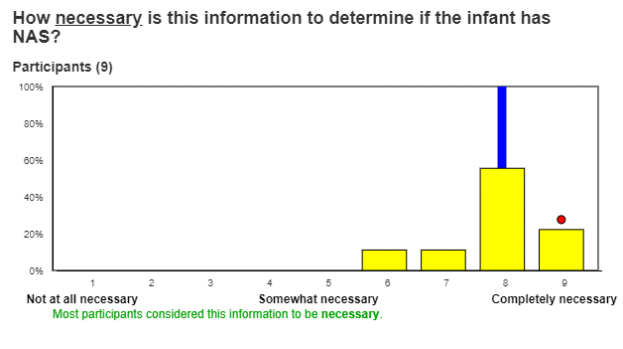
Round 2 mock-up screenshot.

In Round 2, a feedback and discussion round, experts will receive an automatically generated personalized report showing how their individual responses to the rating questions compare to responses of other participants (Figure 3). The report will include a distribution of all responses, a group median response and its IQR, and a statement that explains if the group reached agreement, calculated as described in the RAM manual [22]. The report will also include a summary of comments participants made in Round 1.

Participants will discuss the results of Round 1 using an anonymous, asynchronous, threaded discussion board. To protect confidentiality of participants’ responses, we will use a randomly generated username such as Expert01. We anticipate that this discussion will focus on areas where there may be disagreement or potential confusion among experts. The discussions will be moderated by a clinical expert and a modified-Delphi expert using a previously published protocol for moderating ExpertLens discussions [29]. The same moderators will facilitate online discussions in both panels to ensure consistency.

In Round 3, a reassessment round, experts may choose to revise their Round 1 answers based on Round 2 feedback and discussion or leave them unchanged. Any modifications made to Round 1 questions will be clearly identified within ExpertLens. At the end of Round 3, we will ask experts a series of questions about their experience participating in this ExpertLens process.

We anticipate that each round will be open for 7-10 days, depending on participation rates. At the start of each round, participants will receive invitation emails that will include a description of what they are expected to do in each round, how to access and use the ExpertLens platform, and how long each round will be open. We will send up to three reminders during each round to encourage all participants to provide their input. No honoraria will be offered to study participants.

### Data Analysis

We will use descriptive statistics to present the results of Round 1 and 3 ratings for each panel separately, focusing on the frequency distributions of responses to each question, as well as measures of central tendency (median) and dispersion (interpercentile range). Round 3 rating data will be used for the final identification of suggested definitional components of NAS and NOWS. We will determine the presence of agreement among experts for each rating question using the approach outlined in the RAM manual [22]. Briefly, this approach involves looking at the distribution of responses across the tertiles of scores on the 9-point scale (eg, scores 1-3, 4-6, and 7-9) as a way to explore agreement/disagreement. Disagreement exists when more than a third of responses are in the upper and the lower tertile at the same time. If there is no disagreement, a median of 6.5 and above will indicate a positive group decision.

We will also compare the rating results across the two panels to determine which pieces of information about the infant-mother dyad are necessary and helpful to distinguish between infants with and without NAS or NOWS. These analyses will be important for obtaining expert opinion on the extent to which these two terms differ or overlap, and what pieces of information may be important for developing clinical definitions for each.

To better explain why experts may disagree on which signs are most characteristic of NAS and NOWS, and to explain why some definitional components were selected for inclusion in the definitions of NAS and NOWS while others were not, we will thematically analyze qualitative data, including Round 1 and Round 3 explanations of ratings and Round 2 discussion comments. As in previous ExpertLens panels [30], we will group all Round 1 and 3 comments for a given question according to the score tertile. We will also group all discussion comments by the definitional component. Finally, we will collate and thematically organize the responses to the open-ended questions asking participants to provide their own suggested definitions of NAS and NOWS.

As in previous ExpertLens panels [31,32], a team of experienced qualitative researchers trained by the study’s principal investigators will review and code all comments inductively to identify recurrent themes. All coding results will be reviewed to ensure coding consistency and clinical accuracy of the interpretation of comments. Disagreements among coders will be discussed until consensus is achieved.

### Interpretation, Validation, and Dissemination of Panel Findings

We will provide the advisory board with a synthesis of the quantitative and qualitative data described above. With these data, they will construct a proposed clinical definition of NAS and, depending on the findings from the ExpertLens process, of NOWS as well, using the key pieces of information prioritized by the panelists and drawing on expert input on how to best characterize the variable clinical manifestations of drug withdrawal, which is a major point of debate in the field [33-35].

Results of the online modified-Delphi process and the proposed clinical definitions will be shared with key stakeholders during a national conference on NAS convened by HHS. Participants will be given an opportunity to provide their input. The research team will collaborate with HHS and the advisory board to incorporate input from this national convening in order to refine the proposed clinical definition of NAS, which will be disseminated to key stakeholders, including clinicians, researchers, health system leadership, public health officials, and policymakers.

## Results

The study was reviewed and approved by the RAND Human Subjects Protection Committee (Study ID: 2020-0293). Round 1 invitations were sent out on October 29, 2020. We completed the data collection on December 17, 2020 and disseminated panel results at a national conference on NAS that took place on March 17-18, 2021.

## Discussion

Partnering with experts specializing in maternal-child health, HHS is leading an initiative to understand key issues impacting the longitudinal care of mothers and infants exposed to opioids and other substances. This protocol describes an innovative online approach to soliciting expert opinion, which allows for a nonburdensome engagement of leading national experts on the topic. Study strengths include a focus on a pressing policy issue, a creative approach to data collection during the COVID-19 pandemic, and automatic generation of personalized reports provided to each participant showing how the responses compare with those of other participants and whether or not the panel reached consensus. Study limitations include the engagement of a relatively small number of clinical experts, the possibility that participants may not complete all study rounds or not answer all questions, and a reliance on an online approach to data collection, which may not be a preferred mode of engagement for some experts that are more accustomed to meeting other panelists in person during the discussion round. Because we were able to conduct only one online panel on NAS and one on NOWS, future research should explore the replicability of our findings by conducting additional panels either online or in person.

In summary, a standard clinical definition for NAS is critically needed not only to improve care of the mother-infant dyad in both the short- and long-term but also to enhance the accuracy of surveillance efforts and data available for research. By leveraging input from national experts caring for opioid-exposed dyads, this work will contribute to addressing a longstanding gap in the field: the lack of a standardized clinical definition of NAS. This systematic approach offers a viable pathway for reducing variability in the clinical diagnosis of NAS, starting at the bedside. As an upstream building block for public health surveillance data and health services and health policy research, the bedside definition impacts downstream considerations in the care of mother-infant dyads. Developing a standard to clinically define NAS will advance key discussions on disease burden, immediate and longitudinal needs assessment, and resource planning for mother-infant dyads. In the next phase of this NAS initiative, HHS aims to continue this engagement with clinicians, researchers, and policymakers, while focusing on challenges and opportunities to improve program and policy planning at the local, state, and national levels.

## Appendix

Multimedia Appendix 1 Members of the Advisory Board for the Department of Health and Human Services initiative on neonatal abstinence syndrome.

## Abbreviations

CREDES: Conducting and REporting of DElphi Studies
HHS: U.S. Department of Health and Human Services
NAS: neonatal abstinence syndrome
NOWS: neonatal opioid withdrawal syndrome
RAM: RAND/UCLA Appropriateness Method
