# Supplementary material for: Informing the Development of a Standardized Clinical Definition of Neonatal Abstinence Syndrome: Protocol for a Modified-Delphi Expert Panel
Source: JMIR Res Protoc. 2021 Sep 7;10(9):e25387. doi: 10.2196/25387 (PMC8456327; doi:10.2196/25387)
Supplement: Multimedia Appendix 1 [file resprot_v10i9e25387_app1.docx]

**APPENDIX**

**Members of the Advisory Board for the HHS initiative on NAS**

Jonathan Davis

Matthew Grossman

Lauren Jansson

Hendrée Jones

Stephen Patrick

Mishka Terplan

**Honorary Members of the Advisory Board for the HHS initiative on NAS**

Loretta Finnegan

Karol Kaltenbach
